# Clinical and translational values of spatial transcriptomics

**DOI:** 10.1038/s41392-022-00960-w

**Published:** 2022-04-01

**Authors:** Linlin Zhang, Dongsheng Chen, Dongli Song, Xiaoxia Liu, Yanan Zhang, Xun Xu, Xiangdong Wang

**Affiliations:** 1 Zhongshan Hospital, Department of Pulmonary and Critical Care Medicine, Institute for Clinical Science, Shanghai Institute of Clinical Bioinformatics, Shanghai Engineering Research for AI Technology for Cardiopulmonary Diseases, Shanghai, 200000 China; 2 Suzhou Institute of Systems Medicine, Suzhou, 215123 Jiangsu China; 3 Tsinghua-Berkeley Shenzhen Institute (TBSI), Tsinghua University, Shenzhen, 518055 China; 4 BGI-Shenzhen, Shenzhen, 518083 China

**Keywords:** Biological techniques, Biotechnology

## Abstract

The combination of spatial transcriptomics (ST) and single cell RNA sequencing (scRNA-seq) acts as a pivotal component to bridge the pathological phenomes of human tissues with molecular alterations, defining in situ intercellular molecular communications and knowledge on spatiotemporal molecular medicine. The present article overviews the development of ST and aims to evaluate clinical and translational values for understanding molecular pathogenesis and uncovering disease-specific biomarkers. We compare the advantages and disadvantages of sequencing- and imaging-based technologies and highlight opportunities and challenges of ST. We also describe the bioinformatics tools necessary on dissecting spatial patterns of gene expression and cellular interactions and the potential applications of ST in human diseases for clinical practice as one of important issues in clinical and translational medicine, including neurology, embryo development, oncology, and inflammation. Thus, clear clinical objectives, designs, optimizations of sampling procedure and protocol, repeatability of ST, as well as simplifications of analysis and interpretation are the key to translate ST from bench to clinic.

## Introduction

With the rapid development of biotechnology, scientists coined the term spatiotemporal molecular medicine to understand medicine at multiple dimensions, layers, angles, and dynamics, by integrating spatialization and temporalization of clinical phenomes with spatiotemporal molecular omics.^1^ Spatiotemporal molecular medicine as a new emerging discipline describes pathogenesis, epidemiology, history, patient symptoms and signs, clinical measurements, and therapies. Of those, each has a clear link at four dimensions, e.g., length, width, height, and time. The most important piece of spatiotemporal molecular medicine is to precisely link individual clinical phenomes with molecular changes and to understand the alterations of clinical phenomes based on molecular changes. Spatiotemporal molecular medicine also covers the four-dimensional understanding, diagnosis, and therapy for patients. The spatiotemporal molecular image was conceptualized as the comprehensive integration of dynamics and positioning of molecular profiles by spatial measurements and trans-omics with clinical radiomics and pathological morphology.^2,3^ Of spatiotemporal measurements and analyses, spatial transcriptomics plays an important role in bridging and corresponding information between histological sections and molecular profiles, like cell-cell interplay, transcriptional factor distribution, and spatial location and mRNA expression of the cell, using artificial intelligence, computerized programming, and visualization.

Spatial transcriptome (ST) technologies visualize profiles of RNA molecules in identified tissue regions, including technologies based on micro-dissected gene expression, in situ hybridization, in situ capturing and in situ sequencing technologies.^4^ ST provides the information on transcriptomic expressions and corresponding two-dimensional locations on the tissue at spatial resolution.^5^ The in situ analysis of protein or mRNA in tissue sections is an important part of spatiotemporal molecular imaging. Pathological changes can appear in various positions of the tissue/organ and in different cells within a position dynamically. RNA sequencing of cell bulks fails to clarify the positioning of the altered transcriptomic profiles and spatial distributions of cells. The aims of this present article is to overview the development of experimental and bioinformatic methods of ST and evaluate values of clinical and translational application.

### Invention, development, and history of ST technology

Spatial molecular omics mainly define positional relationship and interactions among cells within the tissue and reveal the impact of spatial cell distribution in the pathogenesis of diseases. The in situ hybridization allows the visualization within original environment by hybridizing with labeled probe complementary to the target molecule. The in situ sequencing (ISS) presents the relationship between cell genotypes/gene expression profiles and morphologic phenomes in local environment.^6,7^ Geographical position sequencing (Geo-seq) analyzes the transcriptome of tissue regions using laser capture microdissection (LCM)^8^ and single-cell RNA sequencing (scRNA-seq), with an accurate resolution as small as 10 single cells and a low throughput.^7,9^ Geo-seq demonstrates the gene expression of morphology-defined cells from specific parts of tissue sections.^4^ ST provides the position information and molecular profiles with high throughput, using spatial barcode microarrays for unbiased mapping of transcripts on entire tissue section.^10^ ST can be combined with scRNA-seq to obtain a comprehensive 3D transcriptome map of the target tissue at single-cell resolution.

**Fig. 1:**
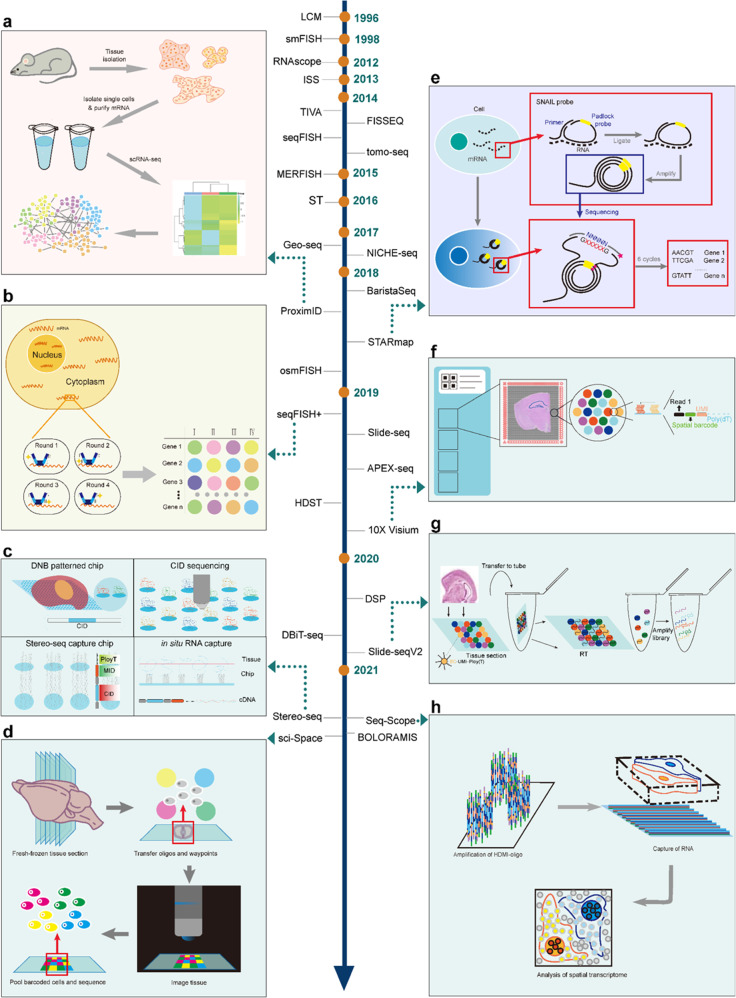
Development of spatial transcriptomic technologies. Representative technologies were exhibited with detailed schematic diagram, including ProximID, STARmap, seqFISH+, 10× Visium, Slide-seqV2, Stereo-seq, Seq-Scope and sci-Space. The development of ST technologies was shown in the middle part with method names and years. **a** The principle from cell isolation to interaction networks for ProximID; **b** The principle of repeated in situ hybridizations for seqFISH+; **c** The principle of Stereo-seq; **d** The principle of sci-Space from fresh-frozen sectioning, oligos and waypoints transferring, and pooled barcoded cell positioning and sequencing, to imaging and reading; **e** The principle of in situ mRNA preparation, SNAIL probe function, and in situ sequencing for STARmap; **f** The principle of in situ capturing from tissue grids to spot selection, from sample setting to quality control, and from partial reads to spatial barcodes for 10× Visium; **g** The principle of Slide-seqV2 from tissue coating to library amplification; **h** The principle of Seq-Scope from high-definition map coordinate identifier (HDMI)-oligo amplification to RNA capture from frozen section to achieve spatial transcriptome analysis at the single cell levels

In the development of ST technologies, representatives include ProximID (Fig. 1a), sequential fluorescence in situ hybridization (seqFISH+) (Fig. 1b), spatiotemporal enhanced resolution omics-sequencing (Stereo-seq) (Fig. 1c), sci-Space (Fig. 1d), spatially resolved transcript amplicon readout mapping (STARmap) (Fig. 1e), 10× Visium (Fig. 1f), Slide-seqV2 (Fig. 1g), and Seq-Scope (Fig. 1h). ST provides new insights into the understanding of embryo development process and defining key genes for specific developing organ, early location, and migration.^11,12^ ST provides better views of cell location and heterogeneity in a developing cell lineage than scRNA-seq per se. Srivatsan et al recently uncovered different functions and differentiations of neurons in locations by combining pseudotime and spatial information.^11^ ST can be of potential assistance for clinical diagnosis and precise treatment customized for the individual.^13,14^ Deep learning algorithm can predict gene expressions in readily available histopathology images after being trained with spatial gene expression and whole-slide histopathology images.^13^ A well-trained model can capture tumor biomarkers, of which some were proven to have clinical diagnosis value. ST with deep learning algorithm could identify new cancer biomarkers, improve efficiency, and accuracy of clinical decisions.

### Technologies for spatially resolved transcriptomics

Micro-dissected gene expression technology provides the spatial gene expression information of captured target regions from samples, by extracting RNA and measuring gene expression with LCM,^15^ RNA tomography for spatially resolved transcriptomics (tomo-Seq),^16^ transcriptome in vivo analysis (TIVA),^17^ NICHE-seq,^18^ or ProximID (Fig. 1a).^19^ LCM can efficiently and accurately define and obtain tissues with target cell subgroups or single cells. Geo-seq clarifies cell heterogeneities and spatial differences simultaneously.^9^ Tomo-seq combines classical cryo-sectioning of tissues with section-based RNA-sequencing and spatially presents transcriptomics with high sensitivity.^20^ Transcriptome maps from tomo-seq can be constructed with the identical biological samples and have challenges to be applied for clinical samples.^16^

**Fig. 2:**
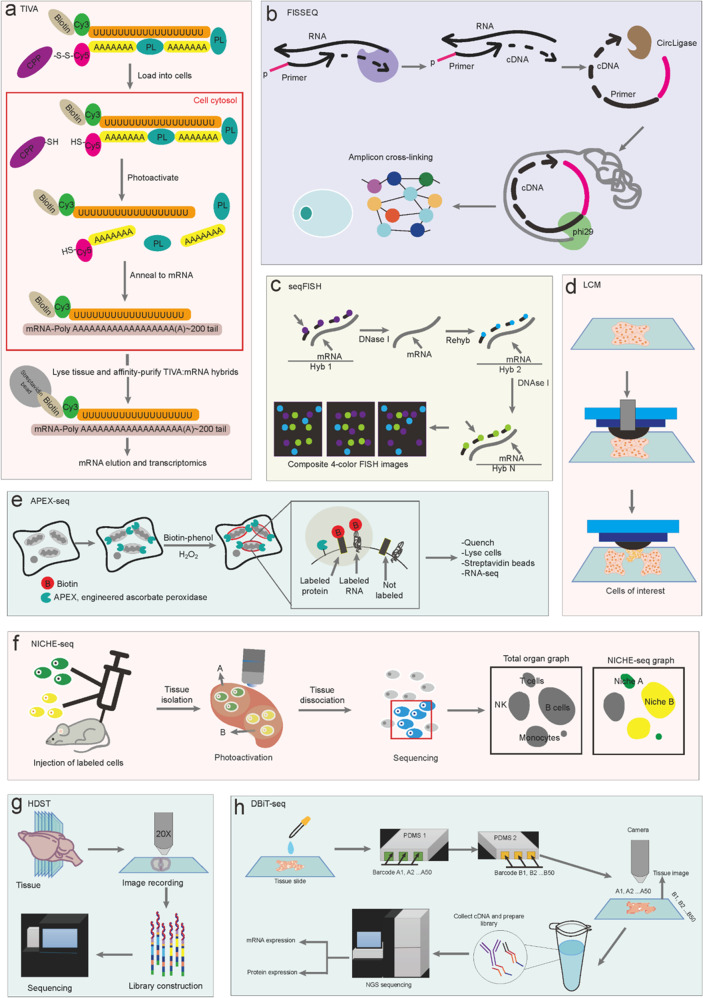
Technical procedures of spatial transcriptomic measurements based on various principles. **a** Procedures from cell loading, photoactivation, mRNA annealing, hybrids, and elution to transcriptomic sequencing TIVA; **b** Procedures from reverse transcription, cDNA linking, circularizing, rolling circle replication, and amplicon cross-linking to sequencing for FISSEQ; **c** Procedures from repeated FISH probe hybridization and digestion to FISH imaging for seqFISH; **d** Procedures from sample coating and selection to capture of target area for LCM; **e** Procedures from biotin-phenol labeling of proteins and genes to RNA-seq for APEX-seq; **f** NICHE-seq includes labeled cells injection, tissue isolation, dissociation, and sequencing of photoactivated cells. **g** Procedures from tissue section, image recording, and library preparation to sequencing for HDST; **h** Procedures from DNA-antibody conjugating, barcoding, tissue imaging, and library construction to sequencing for DBiT-seq

We describe the following ST technologies based on various principles, including TIVA (Fig. 2a), fluorescent in situ RNA sequencing (FISSEQ) (Fig. 2b), sequential fluorescent in situ hybridization (seqFISH) (Fig. 2c), LCM (Fig. 2d), RNA sequencing using the peroxidase enzyme APEX2 (APEX-seq) (Fig. 2e), NICHE-seq (Fig. 2f), high-definition spatial transcriptomics (HDST) (Fig. 2g), and deterministic barcoding in tissue for spatial omics sequencing (DBiT-seq) (Fig. 2h). TIVA is a non-invasive, spatial, and precise method in which oligonucleotide probes are inserted into a live cell (Fig. 2a). It can capture mRNA from living single cells within a microenvironment.^17^ The site-selective, photoinduced biotinylation and mRNA within a living cell has great value in identifying and validating biomarkers, cell types, and responses to therapies in the real-time microenvironment.

NICHE-seq defines rare niche-specific cell subpopulations and transcriptomic profiles within target regions and reconstructs the spatial tissue using photoactivatable fluorescent markers, two-photon laser scanning microscopy, and flow cytometry-based fluorescence-activated cell sorting with scRNA-seq (Fig. 2f).^18^ ProximID can define the interactions and networks among cells, reconstruct the positioning of cell neighborhoods and connections, and visualize ST profiles.^19^ On the other hand ProximID can identify features of cell interactions, however the manual dissection process is labor intensive (Fig. 1a). The value of NICHE-seq and ProximID in reconstruction of spatial cell networks and structure needs to be furthermore validated in more studies and pathological conditions (Table 1; Fig. 2).

**Table 1:** List of representative ST technologies

Launch	Methods	Research group	Institute	Refs
1996	LCM	Lance Liotta	Laboratory of Pathology, National Cancer Institute, Room 2A33, Building 10, 9000 Rockville Pike, Bethesda, MD 20892, USA	^8^
1998	smFISH	Robert Singer	Department of Anatomy and Structural Biology and Cell Biology. Albert Einstein College of Medicine. Bronx, NY 10A61. USA	^21^
2012	RNAscope	Yuling Luo	Advanced Cell Diagnostics, Inc., 3960 Point Eden Way, Hayward, CA 94545	^26^
2013	ISS	Mats Neilsson	Science for Life Laboratory, Department of Biochemistry and Biophysics, Stockholm University, Stockholm, Sweden	^32^
2014	TIVA	James Eberwine	Department of Pharmacology, University of Pennsylvania, Philadelphia, Pennsylvania, USA	^17^
2014	FISSEQ	George Church	Department of Genetics, Harvard Medical School, Boston, MA 02115, USA	^35^
2014	seqFISH	Long Cai	Division of Chemistry and Chemical Engineering, California Institute of Technology, Pasadena, California, USA	^22^
2014	tomo-seq	Alexander van Oudenaarden	Hubrecht Institute, KNAW and University Medical Center Utrecht, Cancer Genomics Netherlands, 3584 CT Utrecht, the Netherlands	^16^
2015	MERFISH	Xiaowei Zhuang	Howard Hughes Medical Institute, Department of Chemistry and Chemical Biology, Harvard University, Cambridge, MA 02138, USA. Department of Physics, Harvard University, Cambridge	^23^
2016	ST	Joakim Lundeberg	Science for Life Laboratory, Division of Gene Technology, KTH Royal Institute of Technology, SE-106 91 Stockholm, Sweden.	^5^
2017	Geo-seq	Naihe Jing	State Key Laboratory of Cell Biology, Shanghai Institute of Biochemistry and Cell Biology, Chinese Academy of Sciences, Shanghai, China	^9^
2017	NICHE-seq	Ido Amit	Department of Immunology, Weizmann Institute of Science, Rehovot, Israel	^18^
2018	BaristaSeq	Anthony Zador	Cold Spring Harbor Laboratory, Cold Spring Harbor, NY 11724, USA	^33^
2018	ProximID	Alexander van Oudenaarden	Oncode Institute, Hubrecht Institute-KNAW (Royal Netherlands Academy of Arts and Sciences), Utrecht, the Netherlands	^19^
2018	STARmap	Karl Deisseroth	Department of Bioengineering, Stanford University, Stanford, CA 94305, USA	^34^
2018	osmFISH	Sten Linnarsson	Division of Molecular Neurobiology, Department of Medical Biochemistry and Biophysics, Karolinska Institutet, Stockholm, Sweden	^25^
2019	seqFISH+	Long Cai	Division of Biology and Biological Engineering, California Institute of Technology, Pasadena USA 911253	^24^
2019	Slide-seq	Evan Macosko	Broad Institute of Harvard and MIT, Cambridge, MA 02142, USA	^38^
2019	APEX-seq	Alice Ting	Department of Genetics, Stanford University School of Medicine, Stanford, CA 94305, USA	^41^
2019	HDST	Patrik Ståhl	Science for Life Laboratory, Department of Gene Technology, KTH Royal Institute of Technology, Stockholm, Sweden	^39^
2020	DSP	Joseph Beechem	NanoString Technologies, Inc., Seattle, WA, USA	^40^
2020	DBiT-seq	Rong Fan	Department of Biomedical Engineering, Yale University, New Haven, CT 06520, USA	^43^
2021	Slide-seqV2	Fei Chen	Broad Institute of Harvard and MIT, Cambridge, MA, 02142	^44^
2021	Stereo-seq	Jian Wang	BGI-Shenzhen, Shenzhen 518103, China	^45^
2021	Seq-Scope	Jun Lee	Department of Molecular and Integrative Physiology, University of Michigan Medical School, Ann Arbor, MI 48109, USA	^46^
2021	BOLORAMIS	George Church	Department of Genetics, Harvard Medical School, Boston, MA 02115, USA	^36^
2021	sci-Space	Cole Trapnell	Department of Genome Sciences, University of Washington, Seattle, WA, USA	^11^

In situ hybridization (ISH) with trackable labels visualizes the gene expressions of specific targets directly in the original microenvironment, including single molecule fluorescence in situ hybridization (smFISH),^21^ sequential single-molecule seqFISH,^22^ multiplexed error-robust fluorescence in situ hybridization (MERFISH),^23^ improved continuous fluorescence in situ hybridization (seqFISH+),^24^ cyclic single-molecule fluorescence in situ hybridization (osmFISH),^25^ and RNAscope.^26^ Labeled nucleic acid probes determine the spatial position and abundance of cellular DNA and RNA within tissues.^22^ Multiple short oligonucleotide probes monitor the expression of the same transcript among various target regions in smFISH.^21,27^ smFISH possesses high sensitivity and resolution of subcellular spatialization, while the spectral overlap under microscope limits the simultaneous detection of many target genes. An advantage of seqFISH, a multiplexed variant of smFISH is that it can detect a single transcript repeatedly via consecutive hybridization rounds, imaging, and probe stripping (Fig. 2c). The number of smFISH probes requires lengthy testing time and vast funds to ensure the number of hybridization rounds.^22^ The coverage of the whole organism by seqFISH staining is highly dependent upon the efficient exchange of macromolecules in dense tissues.^28^

MERFISH, a single-molecule imaging approach, determines copy numbers and spatial localizations of massive RNAs in single cells and combines labeling and continuous imaging to improve the quality and quantity of high-throughput detections by reducing errors of single-molecule labeling and detection with the binary barcodes.^23^ Spatial patterns of gene and genome co-regulations are identified by addressing copy number variation and spatial distribution of genes. The combination of MERFISH with expansion microscopy widens the distance between RNA targets, detects more numbers of RNAs, and reduces spectral overlaps.^29^ The main probes in seqFISH+ contain the flanking area for positioning and are read multiple times to image 10,000 target mRNAs in a cell without super-resolution microscope (Fig. 1b).^24^ Cell types and spatial distributions in tissues are detected with seqFISH+. Multiple transcripts are targeted with fluorescent labels during each hybridization and distinguished by fluorescent colors, after the probe is removed before the next round of hybridization.^25,30^ The number of target genes is highly dependent upon the amount of fluorescence channels and hybridization cycles. OsmFISH with non-barcodes includes certain gene probe sets per round, to overcome the limitation of marker gene numbers and transcript lengths.^25^

RNAscope has two adjacent “Z-probes” with the target sequence and forms the required binding site for further amplification of molecular hybridization, to achieve signal amplification and reduce background.^26,31^ RNAscope is combined with the imaging mass cytometer, to hybridize the DNA tree with metal tags and maximize the capacity of simultaneous detections of RNA and protein targets. This is an important development of individual cell ST analysis, to reconstruct images and positions from co-localization data of biomolecules through chemical reaction and obtain precise genetic information at high spatial resolution.

The subcellular positioning of transcripts can be defined with sufficient signals of amplification for imaging micron or nanometer-sized DNA balls. The in situ sequencing with high specificity and ability identifies targets with single-nucleotide resolution and achieves wide-field imaging. In situ sequencing reduces experimental costs and improves imaging flux, mainly including ISS,^32^ BaristaSeq,^33^ STARmap,^34^ and FISSEQ (Fig. 2b).^32,35^

FISSEQ, a non-target method to capture all kinds of RNA, was launched in 2014, though the sequencing depth was relatively low. FISSEQ is more suitable for identifying cell types based on in situ gene expression profiles.^35^ Using in situ barcode sequencing compatible with Illumina sequencing, BaristaSeq increases the amplification efficiency and sequencing accuracy, suitable for lineage tracing and mapping long-range neuronal projections.

STARmap is another approach of ISS and it hybridizes the target using barcode padlock probe and adds a second primer to the site next to the padlock probe (Fig. 1e). STARmap reduces reverse transcription steps and can overcome efficient barriers of cDNA conversion by adding the second hybridization.^34^ Recently, a reverse transcription-free and in situ RNA identification method, BOLORAMIS, was designed by optimizing the probe design, to improve detection efficiency and sensitivity and map spatial patterns of cells and gene expression by targeting 96 kinds of mRNA, with more than 90% specificity.^36^ The optimized probe can simultaneously and directly target RNA to distinguish point mutations and target shorter transcripts, such as microRNA. BOLORAMIS is suitable for more cell and tissue types, including human brain organoids.^34^

ST was initially developed in 2016.^5^ The efficiency of the resolution was improved from barcode regions of 100 µm to 55 µm in diameter with “10× Visium” (Fig. 1f).^37^ This improves the detection resolution and saves the time for protocol performance. Slide-seq was further developed in 2019, by attaching regional barcode reverse transcription primers to beads in solution and distributing them on a glass surface.^38^ Slide-seq measures genome-wide expression at high spatial resolution and improves the detection of 3’ transcriptome gene expression in fresh-frozen tissues. As compared with other in situ capturing methods, tissue images obtained using Slide-seq originate from the contiguous tissue section, rather than the tissue section where RNA data is generated.

HDST deposits barcodes into finer 2 µm wells with randomly ordered beads and decode positions by several hybridization processes (Fig. 2g).^39^ The signals captured from HDST are highly specific and conformant with those from bulk RNA-seq. Digital spatial profiling (DSP) analyzes highly multiplex spatial RNAs and proteins.^40^ Photocleavable oligonucleotide tags are applied to quantify the abundance of RNAs or proteins in DSP, with single-cell sensitivity in a customizable region consisting of about 5000 cells. Released indexing oligonucleotides tags are counted by the nCounter system or next-generation sequencing.

APEX-seq is an RNA direct proximity labeling technology. APEX-seq provides spatial and localized information of transcriptome using ascorbate peroxidase (Fig. 2e).^41,42^ The DBiT-seq combines spatial analyses of transcriptome and proteome on one tissue section with the resolution of 10 μm (Fig. 2h).^43^ Slide-seqV2 demonstrated highly sensitive ST sequencing at a near-cellular resolution level (Fig. 1g).^44^

Stereo-seq with sufficient capture area and throughput can define the spatiotemporal dynamics of gene expression in tissues and organisms (Fig. 1c). Stereo-seq develops a large field of transcriptomes on tissue at subcellular levels by combining DNA nanoball chips and in situ RNA capture, with high sensitivity and uniform capture rate.^45^ Seq-Scope, as a spatial barcoding method with a comparable resolution has multiple functions and can detect the distance of about 0.5–0.8 μm between pixels, visualize ST heterogeneity at multiple histological scales, and can map subcellular structure of the nucleus and cytoplasm (Fig. 1h).^46^ In contrast sci-Space analyzes nucleus ST on a large scale (Fig. 1d). The single-cell spatial map of mouse E14 embryos was figured out by sci-Space, including spatial coordinates and transcriptomes of about 120,000 nuclei and spatially expressed genes of cell types.^11^ However, sci-Space can only capture the mRNA in the nucleus and loses cytoplasmic transcript information while also limiting the number of cells captured per slice due to low spot density (Table 1).

### Opportunities and challenges of ST technologies

It is difficult to capture the information in a larger area and resolve spatially expression patterns of tissue-specific genes, using technologies based on micro-dissected gene expression or combination of precision microdissection with bulk RNA-Seq. The combination of LCM, and scRNA-seq described transcriptomes of small samples as being composed of about ten single cells. Bulk RNA-seq of frozen sections dissecting the entire Drosophila embryo shows 3D spatial information of embryo^47^ and while tomo-seq can achieve better spatial resolution,^16^ there are still technical limitations to be overcome before clinical practice. TIVA was applied on fresh living tissue by capturing cell-specific gene expressions through laser photoactivation.^17^ While cell numbers can be analyzed simultaneously the real-time applications for living tissues are relatively limited. NICHE-seq based on light-activated technology captures thousands of specific cells by combining scRNA-seq and the accuracy of information on spatial distribution remains unclear. Based on multicellular aggregates formed after tissue dissociation, ProximID demonstrates the communication between proximal cells,^42^ while the flux is low and costs are high. Thus, challenges of spatial technologies based on micro-dissection are low throughput, high labor cost, and limited capture capabilities and information of a large area.

Since its first report six decades ago, FISH technology has been continuously developed to visualize gene expression in fixed tissues.^48^ Challenges that have yet to be addressed include requiring a large number of fluorophores for probes due to the variability of hybridization characteristics, susceptibility of self-quenching, complication of synthesis, and difficulty of purification. smFISH can detect more in situ expression at subcellular resolution with higher sensitivity, by combining 40 short probes.^30^ The number of targeted genes is sparse due to the inherent limitation of spectral overlap. seqFISH improves the accuracy of smFISH quantification by detecting individual transcripts multiple times,^49,50^ however the cost is still high. MERFISH provides better hybridization, shorter working time, wider distance between RNA targets, and more detectable molecules.^23,29^ seqFISH targets the multiplexing of 10,000 genes with confocal microscope,^24^ while osmFISH has lower multiplex and higher quality capacity of larger tissue areas, as compared with other FISH methods.^25^ RNAscope performs RNA assessments,^51,52^ with low throughput. Thus, the economic cost and operative complexity of FISH techniques are relatively high.

In situ sequencing captures target gene information in tissue space and detects sequence information such as alternative splicing, transcript fusion and single-nucleotide variants. BaristaSeq based on padlock probes and sequencing increases the read length and is applied to cultured cells.^53^ STARmap analyzes tissue samples in 3D by introducing a second primer and a hydrogel 3D, rather than a single 2D layer of cells. More than 1000 genes were targeted in 100–150 µm thick slices of mouse brain tissue.^34^ FISSEQ captures RNAs in cultured fibroblasts and detects more than 8000 genes at subcellular resolution.^35^

Transcript capturing in situ followed by ex-situ sequencing avoids limitations of direct visualization and allows unbiased analysis of the complete transcriptome. The RNA capture efficiency is still a challenge in the improvement of resolution and capture/barcode area. ST barcode area with a diameter of 100 µm allows the resolution at 10 to 40 cell level.^54^ HDST enhances the reading depth of each area by randomly depositing 2 µm-sized beads with barcode reverse transcription primers onto an ordered array.^39^ Slide-Seq and HDST improve the capture sensitivity and assistance for defining localized cell types. The HDST resolution reached 2 μm at tissue samples, where each spot captures about 500 UMI. DSP is highly sensitive and can generate usable data with 60–100 cells,^55^ and can also be used for quantitative protein characterization.^56^ APEX-Seq defines the association of specific subcellular locations of RNA in living cells with corresponding function.^41,42^ DBiT-seq contains three resolutions of 10, 25, and 50 μm, of which each pixel at 10 μm resolution can capture about 2000 genes. Stereo-seq achieves sub-cellular resolution with spot distance at 500–715 nm.

With the wide application of machine learning methods,^57^ the integration of ST and machine learning has improved the interpretability of histopathology and application for clinical decision-making, guide precision medicine-based treatment, and predict prognosis of patients.^58,59^ A new dataset of deep learning algorithms was used to spatially predict local gene expression from histopathological images of breast cancer.^13^ The integration of ST with scRNA-seq or multiplexed immunohistochemistry redefines tumor-associated macrophage subpopulations and biomarkers.^60^ The spatial distribution in tumor microenvironment obtained through multiplexed immunohistochemistry and ST provides better views on inter- and intra-tumoral heterogeneity and deeper understanding of relationship between functions and phenotypes of tumor-associated macrophages.

### Bioinformatics tools of ST

Compared with the clustering method of scRNA-seq, ST needs more comprehensive and integrative considerations on gene expression, spatial location, and histological information. A key analytic step of ST is to cluster spots and identify regions where gene expression profiles and morphological phenomes are spatially consistent. Spatial clustering methods analyze spatial dependency of gene expression and mainly include SpaCell,^61^ SpatialCPie,^62^ ClusterMap,^63^ FICT,^64^ SpaRTaCo,^65^ SC-MEB,^66^ and CCST.^67^ Of those, SpaCell can integrate gene expression profiles and imaging data, obtain the embedding layer by training two autoencoders, connect these into a latent matrix, and then subject it to clustering algorithms for further analysis.^61^ ClusterMap was developed for multi-scale clustering analysis for spatial gene expression, precisely locating RNA molecules into subcellular structures or cell bodies in distinct tissue regions.^63^

Identification of spatially variable genes (SVGs) is important to define the location of cell types. With the increasing resolution of ST, new methods are developed to detect spatially variable genes, e.g., GLISS, SpatialDE,^68^ SOMDE,^69^ trendsceek,^70^ or SPADE.^71^ GLISS reconstructs spatial locations by casting scRNA-seq data into spatial dimension.^72^ SVGs in spatial reference are identified as landmark genes, the Laplacian score of each gene is calculated, and then coherent regions with gene expression are spatially obtained.^72^ SPATA detects SVGs along the axis by defining axis and visualizing gene expression and cell type annotations.^73^

In ST data analysis, the distribution of cell types in each spot can be inferred using scRNA-seq data. A variety of cell-type deconvolution methods have been designed for analyses of spatial data, such as SPOTlight,^74^ spatialDWLS,^75^ DSTG,^76^ DestVI,^77^ or STRIDE^78^ and RCTD.^79^ SPOTlight deconvolutes the information of spatial transcriptomics with scRNAseq data using seeded NMF regression,^74^ and DSTG performs cell-type deconvolution by illustrating convolutional neural networks.^76^ RCTD can robustly decompose cell type mixtures in ST data, thus discovering gene expression variety depending on spatial environment.^79^

For ST data lacking the single-cell resolution, each spot may contain different cell types. The quality of analyses can be improved by referring to the information of neighboring spots or to images with high-resolution information of cell morphology and phenomes. Tools for enhancing gene expression resolution include BayesSpace, and XFuse.^80^ BayesSpace dissects spatial transcriptomics data at sub-spot resolution by adopting a Bayesian statistical method which utilizes spatial neighborhood information.^81^ XFuse infers high-resolution spatial gene expression from histological images by assuming that gene expression and histological images share the same spatial state.^80^

Cell-cell communications regulate diverse biological processes to maintain biological functions and microenvironmental homeostasis of cells within organs/tissues.^82,83^ Ligand-receptor pairs are used to explore the communication between different cell types within the tissue and between distinct cells within the same cell type by scRNA-seq, while ST provides the information on cell-cell communication at spatial level. A number of tools was developed to identify cell-cell communications, including GCNG^84^ and SVCA.^85^ GCNG captures the high-order structure of spatial neighborhood map and the direction of causality in new pairs, including predicting novel pairs of genes involved in signal transduction.^84^ SVCA models the expression of interested genes as a Gaussian process and decomposes both non-spatial and spatial sources of variation.^85^ Collectively, the development of ST tools are dependent upon specific purposes, prerequisites, and analysis requirement (Table 2). The consistence and robustness of results should be evaluated by different tools with similar functions or methods with various parameters. Clues from ST data analysis need to be confirmed by comprehensive experimental methods, in order to draw convincing conclusions. Details of ST online resources and ST research groups are listed Table 3 and Table 1, respectively for further references.

**Table 2:** Representative bioinformatics tools of ST

Tool	Website	Refs
SpatialCPie	https://github.com/jbergenstrahle/SpatialCPie	^62^
ClusterMap	https://github.com/xgaoo/ClusterMap	^63^
FICT	https://github.com/haotianteng/FICT	^64^
SpaRTaCo	https://arxiv.org/abs/2110.04872	^65^
SC-MEB	https://github.com/Shufeyangyi2015310117/SC.MEB	^66^
CCST	https://github.com/xiaoyeye/CCST	^67^
SpaCell	https://github.com/BiomedicalMachineLearning/SpaCell	^61^
GLISS	https://github.com/junjiezhujason/gliss	^72^
SpatialDE	https://github.com/Teichlab/SpatialDE	^68^
SOMDE	https://github.com/WhirlFirst/somde	^69^
trendsceek	https://github.com/edsgard/trendsceek	^70^
SPADE	https://github.com/NVlabs/SPADE	^71^
SpatialDWLS	https://github.com/rdong08/spatialDWLS_dataset	^75^
DSTG	https://github.com/Su-informatics-lab/DSTG	^76^
DestVI	https://github.com/YosefLab/scvi-tools	^77^
STRIDE	https://github.com/DongqingSun96/STRIDE	^78^
SPOTlight	https://github.com/maciejkula/spotlight	^74^
BayesSpace	https://bioconductor.org/packages/release/bioc/html/BayesSpace.html	^81^
XFuse	https://github.com/ludvb/xfuse	^80^
GCNG	https://github.com/xiaoyeye/GCNG	^84^
SVCA	https://github.com/damienArnol/svca	^85^
RCTD	https://github.com/dmcable/RCTD	^79^

**Table 3:** Representative ST online resources

Tools	Website	Refs
SpatialDB	https://www.spatialomics.org/SpatialDB	^143^
Allen Brain Atlas	https://portal.brain-map.org/	^144^
eGastrulation	http://egastrulation.sibcb.ac.cn.	^145^
iCHTatlas	https://www.picb.ac.cn/hanlab/ichtatlas/	^146^
EMAGE	http://www.emouseatlas.org/emage/	^147^
eMouseAtlas	https://www.emouseatlas.org/emap/home.html	^148^
BEST	http://best.psych.ac.cn/	^149^

### Application potentials of ST in development and disease

**Fig. 3:**
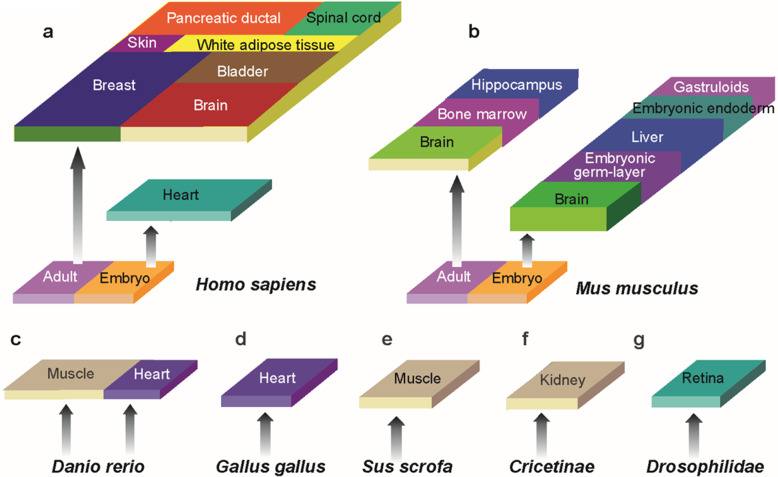
Representative spatial transcriptomics studies across multiple species. **a** Representative ST studies in tissues of *Homo sapiens*; **b** Representative ST studies in tissues of *Mus musculus*; **c** Representative ST studies in tissues of *Danio rerio*; **d** Representative ST studies in tissues of *Gallus gallus*; **e** Representative ST studies in tissues of *Sus scrofa*; **f** Representative ST studies in tissues of *Cricetinae*; **g** Representative ST studies in tissues of *Drosophilidae.*

ST is mainly applied to reveal comprehensive cellular connectomes, sophisticated regulatory networks among distinct cell types, cellular heterogeneity, and microenvironmental homeostasis in the fields of neural science, embryo development, and pathology in *Homo sapiens* (Fig. 3a) and *Mus musculus* (Fig. 3b). In addition, ST profiles of some tissues were mapped in *Danio rerio* (Fig. 3c), *Gallus gallus* (Fig. 3d), *Sus scrofa* (Fig. 3e), *Cricetinae* (Fig. 3f), and *Drosophilidae* (Fig. 3g) (Table 4).

**Table 4:** Representative studies of ST on multiple species

Species	Tissue	Health status	Published year	Refs
Human	Bladder	Cancer	2021	^150^
Human	Brain	Alzheimer’s disease	2020	^100^
Human	Breast	Cancer	2020	^13^
Human	Spinal cord	Amyotrophic lateral sclerosis	2019	^95^
Human	White adipose tissue	Healthy donors and patients	2021	^151^
Human	Pancreatic ductal	Pancreatic ductal adenocarcinoma	2020	^14^
Human	Skin	Cutaneous squamous cell carcinoma	2020	^106^
Human	Fetal heart	Normal	2019	^104^
Mouse	Brain	Alzheimer’s disease	2020	^100^
Mouse	Brain	Alzheimer’s disease	2020	^99^
Mouse	Bone marrow	Normal	2019	^152^
Mouse	Hippocampus, neocortex	Normal	2020	^44^
Mouse	Cerebral cortex	Normal	2021	^153^
Mouse	Gastruloids	Normal	2020	^103^
Mouse	Embryonic liver	Normal	2021	^154^
Mouse	Embryonic endoderm	Normal	2019	^155^
Mouse	Embryonic germ-layer	Normal	2019	^145^
Mouse	Embryonic brain	Normal	2021	^11^
Zebrafish	Muscle	Normal	2021	^156^
Zebrafish	Heart	Normal and *hapln1a* mutants	2021	^157^
Pig	Muscle	Normal	2021	^158^
Chicken	Heart	Normal	2021	^12^
Fruit fly	Retina	Normal	2019	^159^
Hamster	Kidney	Cell line	2018	^33^

ST was widely used to analyze the molecular spatial structure of tissues and to create an atlas of biomolecules in clinical and biological research (Fig. 6a; Table 5).^4^ Using Slide-seq technology, spatial gene expression patterns of mouse and human test subjects were captured and generated with a single-cell resolution.^86^ STARmap analyzed the complexity of 3D brain space with 23 cell type markers in more than 30,000 cells in the primary visual cortex of mice.^34^ The embryonic development of organisms is a complex and dynamic process, of which the expression blueprint and distribution was drawn by ST.^4,87^ Another important application of ST is to study intra-tumoral heterogeneity for precise understanding of tumor progression and treatment outcome. Tumor heterogeneity was re-stratified in prostate cancer by analyzing gene expression gradient within tumor microenvironment in the stroma adjacent to tumor area .^88^ Transcriptomes of nearly 2200 tissue domains were studied in melanoma through lymph node biopsy, to visualize the transcription landscape within the tissue and to identify gene expression profile in a specific tissue area.^89^ ST combined with machine learning identified diagnostic biomarkers from breast cancer ST data, to distinguish ductal carcinoma in situ from invasive ductal carcinoma.^81^ The prediction accuracy of ductal carcinoma was 95% and invasive ductal carcinoma was 91%. The recent ST retains the tissue structure and reflects the immune response by analyzing cell-cell interactions.^90^ Rapid and accurate identification of drug-resistant clones and spatially sensitive biomarkers are needed in cancer patients during targeted therapy, to predict the response to immunotherapy.^91^ ST will provide opportunities for the early detection of gene expression and cellular interactions within the tissue, however precisive resolution and sensitivity of cells for precision oncology need to be furthermore improved.

**Table 5:** Representative ST research on human samples

Sample	Disease	Refs
PDAC tumors tissue	Pancreatic ductal adenocarcinoma	^14^
Intestinal samples	Inflammatory bowel disease/ colorectal cancer	^105^
Prostate cancer tissue	Prostate cancer	^88^
Heart tissue	Angina pectoris	^109^
Heart tissue	Embryonic cardiac samples	^104^
Skin tissue	Cutaneous SCCs\normal adjacent skin	^106^
Pulmonary tissue	SARS-CoV-2, pH1N1 patients, and uninfected patients	^127^
Dorsolateral prefrontal cortex	Postmortem samples	^92^
Gingival tissue	Periodontitis	^108^
Skin tissue	Cutaneous malignant melanoma	^89^
Spinal cord	Amyotrophic lateral sclerosis (ALS)	^95^
Synovial tissue	Arthritis	^10^
Brain tissue	ALS	^96^
Skin tissue	Human leprosy granulomas	^128^

### ST in neurology

**Fig. 4:**
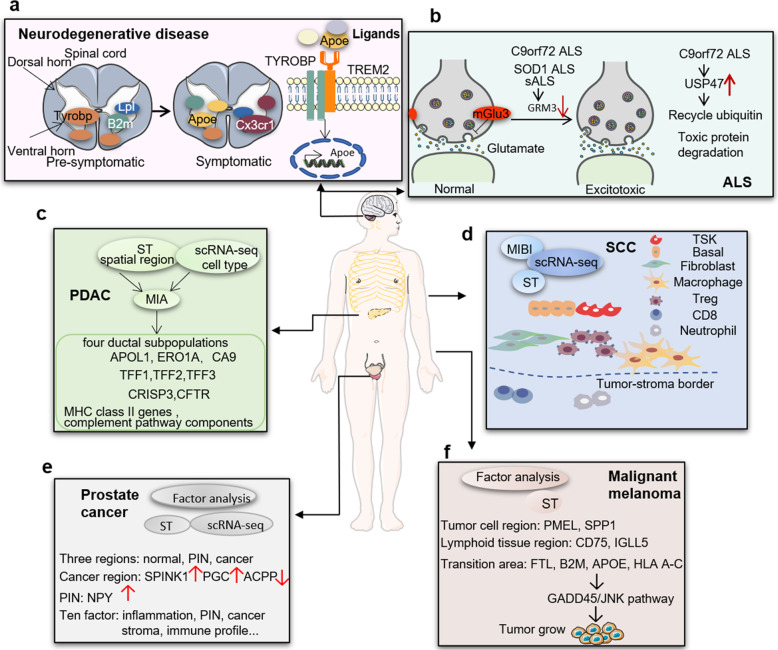
Spatial transcriptomics provide new insights for understanding molecular mechanisms of human diseases and preclinical disease models. **a** In neurodegenerative disease models, *Trem2* and *Tyrobp* form a receptor complex that can trigger phagocytosis or regulate cytokine signaling, when bound by membrane lipids or lipoprotein complexes. *Tyrobp* expression is up-regulated before symptoms and before *Trem2* expression in the ventral horn and white matter. *Lp1* and *B2m* are up-regulated before symptoms, especially in the ventral horn. *Apoe* and *Cx3cr1* are up-regulated in the spinal cord of symptomatic mice. *Apoe* expression is driven by Trem2 signal and the ligand of Trem2. **b** In amyotrophic lateral sclerosis (ALS) models, expression of *GRM3* gene in the prefrontal lobe and motor cortex was lower in *C9orf72* repeat expansion, mutant *SOD1*, and sALS. The *GRM3* gene encodes mGlu3, a metabotropic glutamate receptor, regulates the neurotransmission of glutamate in the central nervous system. The expression of neuronal mGlu3 receptor is mainly at presynaptic terminals. When the extrasynaptic glutamate overflows excessively, the G protein signaling cascade is activated to regulate the activity of presynaptic ion channels, followed by negatively regulating the release of presynaptic glutamate. The regulation that the decrease of mGlu3 receptor expression in the prefrontal cortex increases the transmission of glutamate and produces excitotoxicity may be a common mechanism feature between schizophrenia and ALS. **c** Spatial positions of different subgroups are identified and mapped within cell type in the entire tissue by integrating scRNA-seq and MIA. A total of four ductal subgroups are identified, including ductal population, terminal ductal population, centroacinar duct population, and antigen-presenting duct cells, respectively expressing *APOL1*, *ERO1A* and *CA9* genes; *TFF1*, *TFF2* and *TFF3*; *CRISP3* genes; *CD74*, *HLA -DPA1*, *HLA-DQA2*, *HLA-DRA*, *HLA-DRB1* and *HLA-DRB5*, and *C1S*, *C4A*, *C4B*, *CFB* and *CFH* genes. **d** The combined application of spatial transcriptomics, scRNA-seq, and MIBI demonstrates that TSK cells and basal tumor cells are located on the leading edge. Fibroblasts, macrophages and Tregs are most abundant at the tumor-stroma boundary, while CD8 T cells and neutrophils are largely excluded from the tumor, indicating that the localization of Tregs may prevent effector lymphocytes into the tumor. **e** The gene expression in 3 regions obtained by factor analysis is applied for identifying region-specific markers in normal, cancer and PIN region. Enrichment of *SPINK1* and *PGC*, the depletion of *ACPP*, and the increase of *NPY* level in the PIN area are observed in cancer areas. In addition, the interaction between factors was determined by hierarchical clustering of ten factors. These ten factors include normal glands signature, normal glands, stroma, inflammation, PIN, cancer, immune profile, proximity to PIN signature, and mix of prostatic atrophy and stroma et al. **f** In malignant melanoma models, *PMEL* and *SPP1* overexpressed in tumor cell clusters, and the lymphoid tissue regions from and adjacent to tumor cell regions were characterized by the expression of immune-related genes *CD74* and *IGLL5*, respectively. *FTL*, *B2M*, *APOE* and HLA-related genes (*HLA A-C*) express in the transition zone and related to tumor growth regulation through the GADD45/JNK pathway

The neural system ST provides new insights into understanding individual neuron types, locations, dendritic structures, axon projections, and corresponding functions.^92^ ST profiles of dysfunctional brains offer molecular information on developmental or degenerative organization and location of brain cell types, to develop new molecular biomarkers/targets and to understand new mechanisms of brain diseases.^93^ Paralysis in amyotrophic lateral sclerosis (ALS) is caused by denervation of skeletal muscle, degeneration of motor neurons, dysfunctional interaction between motor neurons and glial cells, and loss of motor neurons.^94^ Using Fiji “Analyze particles” plugin and ST pipeline for data processing, ST with a hierarchical generative probabilistic model was used to measure gene expressions of mouse spinal cords and postmortem tissues of patients with ALS, distinguish regional differences between early microglia and astrocytes, and to identify interferences of transcription pathways between ALS model and patient spinal cord pathology,^95^ as explained in Fig. 4a. Spatial distributions of about 11,138 genes were uncovered in mice dynamically after the induction of ALS and further 9624 genes spinal cord samples were uncovered from patients with ALS. This particular study defined the clear positioning and multi-dimensional distributions of gene expressions in tissue and described new mechanisms of the degenerative disease, e.g., TREM2-TYROBP complex, *Apoe*, *Lpl*, *B2m*, and *Cx3cr1* at different stages and positions.^95^ The 16 dysregulated transcripts in 6 disease-related pathways were found from ST profiles of post mortem brain tissue using ST pipeline for data processing. Of those, the metabotropic glutamate receptor 3 and ubiquitin specific protease 47 were furthermore identified with complementary molecular pathology as spatial disease-specific targets.^96^ The dysregulation of glutamate neurotransmission and synaptic plasticity by the metabotropic glutamate receptor 3 and cell growth and genome integrity by ubiquitin specific protease 47 can be a new alternative of clinical therapies (Fig. 4b).

The combination of ST and scRNA-seq is a new approach to understand the molecular mechanisms of diseases, both dynamically and multi-dimensionally and to develop new classes of spatial biomarkers.^97^ The combination was recommended as an efficient way to diagnose diseases, monitor the progression and therapeutic effects, and to discover new categories of spatial target-based drugs. This combination can feature transcriptional diversities and subclasses of brain endothelial cells in vascular cavernomas, and define functional roles in the development of cerebral cavernous malformation.^98^ It was initially uncovered that angiogenic venous capillary endothelial cells and resident endothelial progenitors might be the major source of cerebral cavernous malformation transformation, rather than arterial endothelial cells. Alzheimer’s disease (AD) is a destructive neurological disease with a gradual loss of mental skills, cognition, and physical functions. AD-associated genes were identified from ST profiles of mouse sections, including spatially dysregulated genes associated with stress responses and mitochondrial dysfunction.^99^ In AD model, ST demonstrated transcriptional changes of co-expression networks rich in myelin sheath and oligodendrocyte genes in tissue section at a diameter of 100 microns around amyloid plaques at the early stage, while in complement system, oxidative stress, lysosome, and inflammation at the late stage.^100^ Spatially defining features and molecular atlas of ST brain regions by unbiased identification could interpret the structure-function relationship of circuits and behavior and the systematic classification of an adult mouse brain.^101^ The annotation of spatial genes in different brain regions provides new insights for understanding the neuron structure, neural connections, interneuron projections, pre-synapses, and glial interactions.^92^ The spatial organization of adult mouse brain regions includes molecular codes for mapping and targeting discrete neuroanatomical domains.^101^

### ST in embryo development

**Fig. 5:**
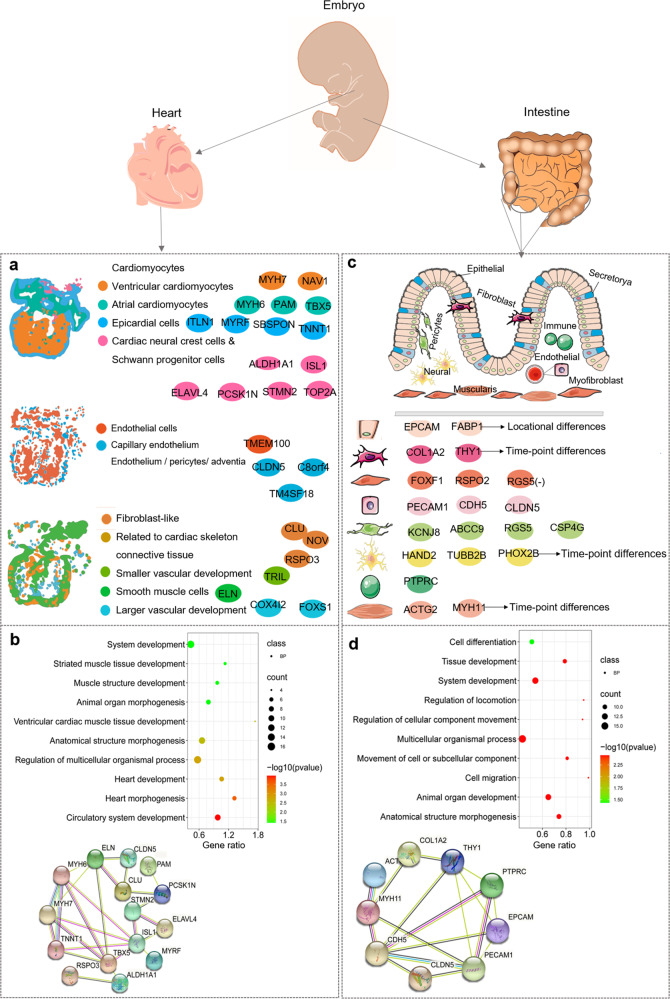
Spatial transcriptomics with scRNA-seq present new cell types and molecular markers in human embryonic development. **a** scRNA-seq analysis of cardiac embryonic tissue shows three types cardiomyocytes (cardiac neural crest cells & Schwann progenitor cells, epicardial cells, ventricular and atrial cardiomyocytes), two types of endothelial cells (capillary endothelium, endothelium/pericytes/adventia) and four types of fibroblast-like cells (related to cardiac skeleton connective tissue, smaller vascular development, smooth muscle cells, larger vascular development). Spatial transcriptome combined with scRNA-seq reveals location distribution and gene markers of each cell type. Cell types and genes are screened based on the reference,^104^ combined with differentially expressed genes in scRNA-Seq clusters and spatially heterogeneous gene panel. **b** GO (BP: biological process) analysis of heart cell type-specific genes and protein interaction networks (https://cn.string-db.org/). **c** Spatiotemporal analysis of human intestinal development at single-cell resolution identify nine intestinal compartments including epithelial, fibroblast, endothelial, pericytes, neural, muscularis, mesothelium, myofibroblast, and immune cells. Each type cells have specific gene markers, of which some have location and time-point differences in gene expression. *FABP1* expresses specifically in epithelial cells and over-expresses in colon. *HMGA2*, *MYH11*, and *PHOX2B* down-express consistently in colon and terminal ileum. Intestinal cell signature genes are identified according to Supplemental Table 1 of the reference,^105^ which exhibit specific key genes expression in each cell types. **d** GO (BP: biological process) analysis of intestinal cell type-specific genes and protein interaction networks (https://cn.string-db.org/)

Embryos and stem cell lineages serve as paradigms to explore the tissue patterning and corresponding molecular regulation circuits. ST with scRNA-seq have opened up new ways for dissecting molecular dynamics of cell organization, differences in morphology and molecular properties, and lineage allocation during the process of embryo development.^102^ The process of somitogenesis was explored for key features of embryos and development after implantation in mouse gastruloids using ST with scRNA-seq.^103^ The comprehensive transcriptional landscape of human embryonic heart cell types, cell-type distribution, and spatial organization reveals three developmental stages at 4.5–5, 6.5, and 9 post-conception weeks.^104^ Using Seurat package for data processing, ST profiles of three human embryonic cardiac samples showed about 3,115 individual spots and 10 myocardial clusters during development of human embryonic heart.^104^ Spatial maps of cell types, transcriptomes, and spatiotemporal maps of human heart development were obtained from about 69 selected target genes (Fig. 5). scRNA-seq analysis of cardiac embryonic tissue yielded three-types cardiomyocytes, two type endothelial cells, and four-type fibroblast-like cells. By combining ST, scRNA-seq, and GO analysis it enabled a better identification for location distributions and gene markers of each cell type in the tissue (Fig. 5a), heart cell type-specific genes, and interaction networks (Fig. 5b). Such mapping of the human embryonic heart allowed scientists to fully exploit valuable resources, cell-cell communication, and lineage development. By using 10× Genomics Visium and Space Ranger software, ST with scRNA-seq demonstrated the spatiotemporal atlas of human intestinal development and morphogenesis.^105^ In this particular study, 77 intestinal samples were collected from 17 embryos representing different developmental time points and tissue locations, and 101 subgroups were identified in the compartment based on the fine cluster annotation of key marker genes. Transcriptional regulatory networks were positioned and exampled in Fig. 5c, to highlight the key regulatory network of each cell type and to reconstruct the “decision tree” of cell fate using scRNA-seq, including known developmental regulators, 306 developmental time courses, and 44 positional changes of regulatory networks. ST analysis of tissues from the entire intestinal development (Fig. 5c) revealed spatiotemporal locations of scRNA-seq-identified clusters, formation of epithelial crypt villi, differentiation of mesenchyme, establishment of muscle layer, expansion of vascular system, and immune colonization and appearance of gut-associated lymphoid tissue.^105^ Each type of cells has specific gene markers, of which some have location and time-point differences in gene expression (Fig. 5c), intestinal cell type-specific genes, and interaction networks (Fig. 5d). The outstanding findings from this particular study provides new insights for understanding neonatal diseases and genetic defects of intestinal development (Table 5).

### ST in pathology

ST-characterized 3D images present the heterogeneity of interaction between tumor and immune cells and the difference of infiltrated immune cells between locations.^106^ Using machine learning models, capacities of analysis, description, and stratification of interaction between tumor and immunity were improved with high accuracy.^107^ ST of melanoma lymph node biopsies revealed the heterogeneity of transcriptional landscape and gene expression profiles among spatial intra-tumoral compositions linked with histological entities^89^. The gene expression pattern of the lymphoid area in close proximity to the tumor region was similar to that of tumor microenvironment. Spatial positioning of transcriptomic profiles has great potential to help us understand molecular mechanisms of tumor progression, metastases, and to identify therapeutic targets. ST profiles of periodontal tissues showed up-regulated expression of specific polar shadow in the inflammatory area as part of pathogenesis of chronic inflammatory periodontitis.^108^ ST profiles of adult heart demonstrated spatial differences of fetal gene expression among patients with heart failure.^109^

Intra- and inter-tumoral heterogeneities are highly associated with the expansion of tumor subclones with genetic, genomic changes, and interactions between tumor cells in the tumor microenvironment and are responsible for tumor progression and complexity.^110^ ST profiles of various diseases were mapped, including within brain tissue,^92^ spinal cord tissue,^95^ breast cancer,^5^ cutaneous malignant melanoma,^89^ prostate cancer,^88^ gingival tissue,^108^ pancreatic tissue,^14^ and human heart tissue.^109^ Moncada et al. performed scRNA-Seq and ST (custom-built pipeline for data processing) analysis in two tissue samples of primary pancreatic ductal adenocarcinomas and developed a multimodal intersection analysis to determine the degree of overlapping genes, as compared with accidentally expected analysis (Fig. 4c).^14^ This serves as initial evidence to uncover subpopulations of tissue cells across spatial regions, functional interactions among cell types, and stress-associated re-distributions of inflammatory fibroblasts with cancer cells. The combination of scRNA-seq, ST, and multiple ion beam imaging revealed the main locations of tumor-specific keratinocytes (TSK) populations at the leading edge of human cutaneous squamous cell carcinoma.^106^ TSK cells recruit specific cell types like Treg cells to cancer sites by integrating ligand-receptor networks as the hub of cell-cell communication and express chemoattractive factor genes, while CD8 T cells and neutrophils are absent in the tumor microenvironment (Fig. 4d). This indicates that Treg re-localization may be one of potential mechanisms by which the infiltration of effector lymphocytes is prevented into tumor. Patients with high expression of TSK markers, integrin-beta1, and urokinase-type plasminogen activator, had lower progression-free survival rates after receiving PD-1 checkpoint inhibitor.^106^ This is an example to apply ST for understanding tumorigenesis and progression, formation of tumor microenvironment, and therapy in the complex ecosystem. It should be furthermore clarified what the number of patients, samples per patient, and sections per sample are enough to make a conclusion from ST. In a prostate cancer study, the conclusion that the spatial pattern was correlated with the histologically identifiable structure was made on basis of patient sample including normal, cancer, and prostatic intraepithelial neoplasia areas, using the Poisson factorization core model of factor analysis (Fig. 4e).^88^ This analysis was used in ST study on cutaneous malignant melanoma,^89^ firstly delineating cell-type-specific and tissue-region-specific genes and overlap (Fig. 4f). Of those four studies,^14,88,89,106^ three were analyzed with the combination of ST and scRNA-seq by integrating two datasets.

Spatiotemporal molecular pathology is a ST-based concept and a critical part of spatiotemporal molecular medicine, including multi-dimensional morphological phenomes, intracellular organelle functions, and gene/protein interactions.^111–115^

Spatial transcriptome has important application value in analyzing the pathogenesis of infectious diseases. The integration between scRNA-seq and ST of ileal and cardiac tissues from neonatal mice with reovirus infection at different time points revealed the dynamic process of myocarditis infection pathogenesis, the spatially heterogeneous network of different cellular phenotypes, and the association with virus-induced intercellular interactions.^116^ Spatiotemporal multi-omics and trans-omics will provide even more important and comprehensive insights for understanding the disease. More measures are needed to define quantitation, spatiotemporal localization, mutations, splice isoforms, and posttranslational modifications variants of expressed target proteins.^114^ The application of spatiotemporal techniques to clinic is still lagged behind by complicated procedures of sample preparation, quality control, data generation and interpretation, or high cost.^117^ The novelty and standardization of methodologies should be furthermore developed, e.g., enhanced throughput (number of spots or cells captured per experiment) and augmented resolution (number of genes detected per cell). The spatial trans-omics is a new direction of ST development and requires simultaneous detections of chromosome structure, chromatin accessibility, histone modification, DNA methylation, transcriptome, proteome, metabolism, and non-coding RNAs in one sample. Tools based on cloud computing and artificial intelligence will allow scientists to interpret complicated spatiotemporal data easily and freely. With the rapid development of sequencing methods, library construction protocols, and chemical reagents, the cost will be reduced, efficiently making spatiotemporal molecular pathology a candidate for clinical screening, diagnosis, and therapeutic monitoring.

### ST in inflammatory diseases

Spatial locations or natural states of cell clusters and subtypes as well as corresponding genes in tissues represent different signal concentration gradients, according to external stimuli, developmental directions, stages of tumor metastasis, and “interface” cell states in tumor microenvironment.^118^ The spatial cell-cell communication in microenvironment provides more detail information on biological functions, networks, and host-pathogen interactions in development of organs and diseases. Circulating biomarkers of patients with COVID-19 or pandemic H1N1 influenza were measured, although it is still questioned whether parameters in peripheral blood can accurately reflect the viral load or degree of tissue damage.^119–121^ The ‘bulk’ sequencing of whole lung tissue hardly describes the heterogeneity and positioning of infected cells.^122,123^ The spatial information of tissues provided deeper understanding of cell changes and characteristics of transcriptomic profiles in response to viruses, although the number of genes differentially expressed between the lungs of influenza and COVID-19 patients was limited^124^ and it remains unclear how to translate the knowledge of COVID-19 into the prevention of Omicron variants.^111,125,126^ Genes related to inflammation, type I interferon production, coagulation, and angiogenesis were up-regulated in lungs of patients with COVID-19. ST profiles of COVID-19 infected lungs presented novel gene signatures closely associated with the pathogenesis of SARS-COV-2.^127^ SARS-CoV-2 was unevenly distributed in lungs, with few areas of high viral load in responses to increased type I interferon.^127^

ST describes immune cell mechanisms and tissue diversities caused by chronic inflammatory diseases, including over-expression in*CD3E*, *CXCL9*, *CXCL13*, and *LTB* in rheumatoid arthritis. While *POSTN*, *COMP*, *CILP2*, and *PRG4* in spondyloarthritis were related to higher cartilage turnover rate.^10^ The cell-cell interaction network and spatial location of gene expression is critical in the progression of inflammation. Study on human leprosy granulomas demonstrated that the construction of molecular networks revealed the tissue structure of granuloma, constituent and functional layers, and clusters of cells with unique antimicrobial genes and secreted cytokines to promote antimicrobial responses,^128^ including macrophages, T cells, keratinocytes, and fibroblasts. Different from lung cancer, multi-inflammatory factors and cells contribute to the formation of lung microenvironment during infection and inflammation.^112,129–131^ Local hyperresponsiveness and re-distributions of fibroblasts, hyper-production of inflammatory mediators, and over-activation of extracellular matrix proteases occurred in the interstitial inflammation area within mouse lung section using 10× Genomics-based ST.^132^ It is important to define the correlation between tissue ST and scRNA-seq with circulating scRNA-seq and develop scRNA-seq as a routine measurement of clinical biochemistry in clinical decision-making and guide therapy.^133^

### Potential challenges for clinical application

It is important to understand spatial heterogeneity in occurrence and development of diseases and develop ST for defining gene/protein/cell types and precise location in the lesion tissue, since gene expression occurs on complex spatial forms during biological processes.^134^ Precise selection of methods is one of the critical steps to optimally present an atlas of disease-specific molecular interactions. For example, a type of disease-associated microglia was found to be associated with neurodegenerative diseases in AD model using scRNA-seq, while the location of such microglia type near the Aβ plaque and closely related to the origin of AD was determined in human AD postmortem brain using RNA-seq and smFISH.^135^ Increased number of intracellular Aβ particles and positive disease-associated microglia markers were validated using immunohistochemical staining. This discovery provides an opportunity to develop microglia activating drugs for AD-targeted therapy. Although ST shows location-based gene expression phenomes, the regulation of pathways and cell states in differentiation of secretory and ciliary lineages was furthermore explored in endometrial organoids using 10× Genomics Visium technology.^136^ NOTCH and WNT pathways control the mechanism for differentiation efficiency of cilia and secretory epithelial cells and provides an idea for the treatment of endometriosis and endometrial cancer.

**Fig. 6:**
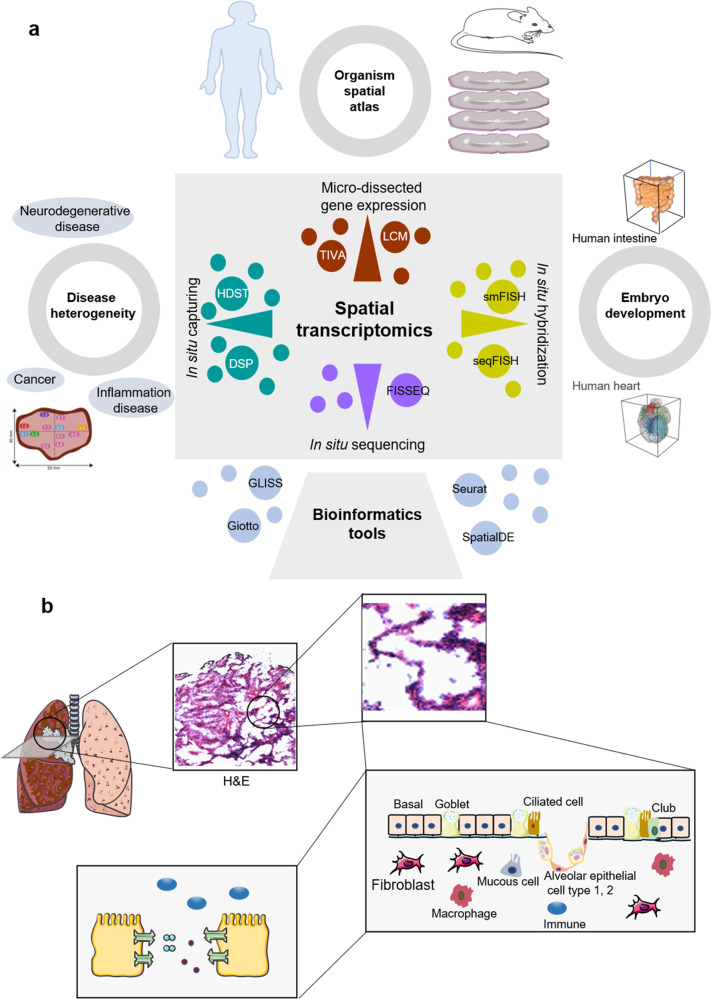
Summary of workflows during applications for spatial transcriptomics. **a** Spatial transcriptomics techniques are mainly used to solve spatial heterogeneity of diseases, biological spatial transcriptome map, and embryonic development spatial blueprint. The four ST techniques include micro-dissected gene expression, in situ hybridization, in situ sequencing and in situ capturing. Different techniques are used according to different sample characteristics and needs, and a variety of biological tools are used for analysis. **b** A simulation diagram of tumor microenvironment. ST technology assists in understanding the spatial location of tumor cells and gene expression as well as intercellular molecular communication between cells within the microenvironment. For example, H&E staining in lung tissue provides sample pathological information and assesses sample quality, and further integrates cell grouping and location information analysis such as basal, goblet, ciliated cell, alveolar epithelial cells type 1, 2 and other cells, so as to provide a better understanding of the molecular communication mechanisms in the cellular microenvironment

ST profiles present 3D molecular phenomes and descriptive information with identity, degree, and location of expressed genes. The spatial expression of fetal genes in adult cardiac biopsy in patients with heart failure were similar to those of fetal myocardium.^137^ Over-expression of fetal marker genes contributed to the remodeling of the human heart.^109^ The hepatic zonation represents the most important factors responsible for spatial heterogeneity and identity of the vascular structure on basis of spatial expression of target genes in the liver lobule zonation.^138^ Preclinical ST studies demonstrate the cell-cell communication and dysfunction between somatic cells of ischemic neurons and axon mitochondria at single-cell level,^139,140^ and between tissue cells in acute kidney injury induced by endotoxemia.^141^ Such spatial patterns of gene expression help us understand the occurrence and development of heterogeneity, including microenvironment, intra- and inter-tumors, reoccurrence, metastasis, and responses to treatment (Fig. 6b). ST profiles provide a clear positioning of gene expression and cell-cell interaction, as compared with scRNA-seq, while both belong to comprehensive descriptive information. Different from scRNA-seq, ST produces the descriptive information on spatial phenomes for the proposal of molecular mechanisms.

The clinical significance and application of ST are highly dependent upon the matchability, reproducibility, and stability of ST profiles and upon disease complexity, severity, stage, pathology, and structural accuracy. Within ST profiles of the multicellular gene regulatory network, 57 plaque-induced genes gradually constructed a regulatory network in AD model.^100^ The deposition of β-amyloid plaques may play a “trigger” or “drive” role in the course of disease, partially as neurodegenerative mechanism of multicellular synergy. It is a challenge to translate preclinical ST profiles into clinical values due to variations of molecular profiles and morphological phenomes between animals and humans and between models and diseases.

The complexity and repeatability of ST with comprehensive analyses are seriously considered as technical limitations for clinical application.^7^ There is growing evidence that ST is an important tool for understanding of human diseases,^102^ monitoring spatial heterogeneity of molecular signals and cell-cell interaction, and developing precision medicine strategy. ST profiles are dependent upon the positioning of histological sections in 3D organs/tissues and upon the dynamical phase of disease progressions. It is questioned whether the degree of ST profiles in selected histological section represents and covers the full landscape and changes of multi-dimensional organs, due to massive variations among tissue sections at different levels and orientations. Molecular features from cancer ST data may classify transcriptomic interactions within breast cancer regions and provide scientific evidence and supports for clinical understanding and decision.^107^ The precision of pathological identification and selection is decisive in reliability and matching of ST profiles and corresponding cell-cell interactions. Although ST profiles have made advances in discovery and identification of disease-specific and spatialization-specific factors, comprehensive analyses for constructing spatial maps and drawing spatial blueprints need to be furthermore standardized, automatized, and characterized for large scales of ST analysis, reduction of methodological variations, and more potential explorations.^4^ The number of human samples are still limited for clear conclusions due to difficulty of sample collections, preservation, and transports.^117^ There are urgent needs to have systemically well-designed clinical studies on target diseases with clear information on section location, disease phase and severity, and interventions. ST currently impacts our understanding and knowledge about transcriptomic regulations and cell-cell interactions in tissue structure, while the long-term impact of ST should be evidenced by clinical applications of integrated ST with clinical phenomes.

With the rapid development of new ST technologies, data acquisition is constantly being improved and challenges in ST resolution, sensitivity, throughput, and accessibility are being overcome. ST is compatible with paraffin-embedded tissues,^100,142^ providing the possibility for retrospective analysis of samples collected in biobanks. It will be possible to systematically detect a variety of tissues and reconstruct 3D spatial structure gene expression of organisms.^58^ Improved ST will allow us to further understand the development process of organisms and provide the basis for early disease detection in clinical medicine and precise targeted therapy. Combined application of ST with multiple technologies will be needed to meet clinical requirements. Thus, we believe that continuous improvements of ST resolution and sensitivity at single-cell resolution will benefit clinical practice and improve patient outcomes.
